# Post-Fledging Dispersal of King Penguins (*Aptenodytes patagonicus*) from Two Breeding Sites in the South Atlantic

**DOI:** 10.1371/journal.pone.0097164

**Published:** 2014-05-14

**Authors:** Klemens Pütz, Phil N. Trathan, Julieta Pedrana, Martin A. Collins, Sally Poncet, Benno Lüthi

**Affiliations:** 1 Antarctic Research Trust, Bremervörde, Germany; 2 British Antarctic Survey, Natural Environment Research Council, High Cross, Cambridge, United Kingdom; 3 Recursos Naturales y Gestión Ambiental, CONICET - INTA, Balcarce, Argentina; 4 Government of South Georgia & South Sandwich Islands, Stanley FIQQ 1ZZ, Falkland Islands, South Atlantic; 5 Antarctic Research Trust, Stanley, Falkland Islands, South Atlantic; 6 South Georgia Surveys, Stanley, Falkland Islands, South Atlantic; 7 Antarctic Research Trust (Switzerland), Forch, Switzerland; Institut Pluridisciplinaire Hubert Curien, France

## Abstract

Most studies concerning the foraging ecology of marine vertebrates are limited to breeding adults, although other life history stages might comprise half the total population. For penguins, little is known about juvenile dispersal, a period when individuals may be susceptible to increased mortality given their naïve foraging behaviour. Therefore, we used satellite telemetry to study king penguin fledglings (n = 18) from two sites in the Southwest Atlantic in December 2007. The two sites differed with respect to climate and proximity to the Antarctic Polar Front (APF), a key oceanographic feature generally thought to be important for king penguin foraging success. Accordingly, birds from both sites foraged predominantly in the vicinity of the APF. Eight king penguins were tracked for periods greater than 120 days; seven of these (three from the Falkland Islands and four from South Georgia) migrated into the Pacific. Only one bird from the Falkland Islands moved into the Indian Ocean, visiting the northern limit of the winter pack-ice. Three others from the Falkland Islands migrated to the eastern coast of Tierra del Fuego before travelling south. Derived tracking parameters describing their migratory behaviour showed no significant differences between sites. Nevertheless, generalized linear habitat modelling revealed that juveniles from the Falkland Islands spent more time in comparatively shallow waters with low sea surface temperature, sea surface height and chlorophyll variability. Birds from South Georgia spent more time in deeper waters with low sea surface temperature and sea surface height, but high concentrations of chlorophyll. Our results indicate that inexperienced king penguins, irrespective of the location of their natal site in relation to the position of the APF, develop their foraging skills progressively over time, including specific adaptations to the environment around their prospective breeding site.

## Introduction

Integrated knowledge on the spatial distribution of long-lived animals, including all life history stages, are of paramount importance to fully understand their movements and distribution in view of potential population trends, threats and the subsequent implementation of appropriate conservation measures. Almost all information currently available for meso-predators such as sea turtles [1], tunas [2] or seabirds [3] are generally both spatially and temporally patchy with coverage often limited to a small part of the population; e.g. usually adults or breeding animals [2], [4]. In particular, there is generally a widespread lack of information on immature animals [5], even though this demographic category can represent up to half of the total population of some long-lived meso-predator species (e.g. [6]). Moreover, juveniles with their naïve behaviour may have higher susceptibility to increased mortality associated with variability in their environment [7], [8] and/or lack of genetic fitness.

After fledging, young animals generally forage and disperse on their own using a portfolio of learned and/or genetically pre-determined behaviours [9], [10], [11]. During this period, in which naïve animals acquire the skills of efficient locomotion and foraging [10], [12], [13], they may not only have high mortality rates through natural causes [14], but they may also be more susceptible to human-induced threats. The most critical stage, often with a high risk of mortality, usually occurs during the first year when juveniles can disperse over relatively long distances (compared with breeding season foraging trips made by adults) and across completely unknown ocean areas while having to acquire effective foraging skills [15]. The behaviour of seabirds during this period of their early life is almost unknown because of the difficulty of tracking their movements over long periods, especially in the pelagic marine environment [16]. Thus, little if any information is available on this critical period when young individuals disperse from their natal colony [17]. Among Southern Ocean seabirds, only shy (*Thalassarche cauta*) [18], Amsterdam (*Diomedea amsterdamensis*) [19] and wandering (*D. exulans*) albatrosses [7], [10], emperor (*Aptenodytes forsteri*) [16], [20], [21], [22], gentoo (*Pygoscelis papua*) [23] and Adélie (*P. adeliae*) penguins [24] have been tracked as fledging juveniles. Surprisingly, in view of the number of studies performed on emperor penguin juveniles and the two smaller penguin species, nothing is known about the dispersal of juvenile king penguins (*A.* *patagonicus*).

King penguins have a circumpolar distribution [25], [26] and breed on islands located within 400 km of the Antarctic Polar Front (APF). At the APF, cold and nutrient-rich Antarctic waters sink beneath the relatively warmer subantarctic waters, while associated zones of mixing and upwelling create a zone very high in marine productivity [27]. Studies on the king penguin foraging areas during different times of the year have shown that during summer adult birds forage mainly within the vicinity of the APF, irrespective of whether the APF is located north or south of the breeding site ([28], [29], [30], [31], [32], but see also [33]). However, in winter birds forage mostly south of the APF in Antarctic waters, sometimes as far as the northern limit of the pack ice [29], [31], [32], [34]. This change in foraging area is reflected in a shift in diet: in summer penguins feed predominantly on myctophid fish and to a lesser extent on squid; however, the latter component becomes more important in winter times [35], [36]. The only exception from this behaviour has been observed in the Falkland Islands, where in summer birds forage towards the sub-Antarctic Front and in autumn in the vicinity of the APF. In winter, Falkland Island birds forage to the north of the breeding site along the slope of the Patagonian Shelf, which has been attributed to a behavioural adaptation to exploit the highly productive waters in the area [32].

Of the global population of over 1.5 million king penguin breeding pairs [26], about 450,000 breed at South Georgia, making this site the major breeding site in the Atlantic sector of the Southern Ocean [37]. With the exception of isolated cases of egg-laying recorded at other localities [38], [39], the only other breeding site in the area is the Falkland Islands, where a small population of about 720 breeding pairs, recently fledging ca. 500 chicks annually [40], [41], has become established over the past 50 years. Although South Georgia is located only 300 km further south than the Falkland Islands, both breeding sites are located at the distributional limits of king penguins, because the APF turns north as it crosses the northern Scotia Ridge, resulting in the Falkland Islands being located up to 400 km north and South Georgia being located up to 300 km south of this prominent oceanographic boundary.

The aim of this study was to examine the post-fledging dispersal of juvenile king penguins in the South Atlantic using satellite telemetry. Juvenile king penguins were tracked simultaneously from South Georgia and the Falkland Islands to address the following questions: 1) How do juvenile king penguins disperse during their first year at sea with regard to the position of the APF? 2) Do they disperse potentially following a genetically pre-determined behaviour, or do they disperse randomly over the open ocean? 3) Do they use a different habitat from that of adults? 4) Do juveniles from the two breeding sites differ in their foraging habitats? 5) What are the key environmental parameters influencing their distribution?

## Materials and Methods

### Ethics Statements

This study was approved by the Falkland Islands Government (R 09/2007) and the Government of South Georgia and the South Sandwich Islands and complied with the legal requirements in the United Kingdom. The king penguin colony in the Falkland Islands is located on private land (Johnson’s Harbour Farm); permission was given by the owners Osmund and Olive Smith (today: Jan Cheek, Stanley, Falkland Islands). King penguins are not endangered (classified as ‘Least concern’ in the current IUCN red list) but are protected under the ‘Conservation of Wildlife and Nature Bill 1999’ in the Falkland Islands and under the South Georgia Environmental Management Plan in South Georgia [26]. The procedures used in this study were scrutinised and approved by the Animal Ethics Committee of the British Antarctic Survey. The greatest care was taken to minimize stress while handling animals, which lasted less than 20 min in all cases.

### Study Site and Device Attachment

Fieldwork was conducted in the king penguin breeding colonies located at Volunteer Beach (51°29′S, 57°50′W), Falkland Islands, and at St. Andrews Bay (54°27′S, 36°11′W), South Georgia, on 11 December 2007 and 13/14 December 2007, respectively. King penguin fledglings were randomly selected at each site (Falkland Islands n = 10, South Georgia n = 8) After their capture, penguins were weighed (South Georgia only; mean body mass 9.5±1.2 kg) before satellite transmitters were attached using black waterproof tape (Tesa, Beiersdorf AG, Hamburg, Germany) and glue, adapted from the methods described by [42]. Devices were hydrodynamically shaped in order to minimise drag [43], [44] and centered on the middle of the back in order not to compromise the penguins’ balance [45] or create excessive hydrodynamic drag. The devices were finally covered with a layer of quick epoxy (Loctite 3430, Loctite Deutschland GmbH, München, Germany) to prevent the birds from removing the tape with their beaks. All birds were assigned names chosen by the individual funding sponsors.

The KiwiSat 202 satellite transmitters used had maximum dimensions of 80×35×27 mm and weighed approximately 60 g, equivalent to about 0.6% of the mean penguin body mass. The flexible antenna of each device was 185 mm long and had a diameter of 2 mm: it originated with an angle of 60° at the rear of the device to further reduce drag [46]. To reduce the energy requirements of the satellite transmitters, devices were programmed to transmit with a repetition period of 60 s and with a duty cycle of 4 hours on/20 hours off. Devices were powered by 2×AA cells, providing a maximum life span of 240 days. All transmitters were switched on at 01∶00 GMT ( =  local time +3 hours at the Falkland Islands, and local time +2 hours at South Georgia), because penguins are optically orientated predators and thus more likely to be less active at night [47], thereby increasing the likelihood of successful transmissions while the penguin was resting at the sea surface.

Positional data obtained from Argos (CLS, Toulouse, France) were classified according to the quality of the positional fix, with location classes 0, 1, 2 and 3 representing accuracies of >1 km, <1 km, <350 m and <150 m, respectively [48]. Only the most accurate position obtained from each duty cycle for each penguin was processed as the ‘daily position’ (98.6% of which were accurate to within 1 km or better). Based on these filtered positions, the following migratory parameters were calculated: Maximum distance to the colony ( =  furthest distance to the natal site during the tracking period), minimum distance covered ( =  sum of distances between consecutive positions), mean and maximum daily distance covered.

### Habitat Modelling

Presence/absence modelling requires the definition of a grid of spatial units in which the presence or absence of the species is recorded. We applied different methodological approaches to identify different marine habitats used by king penguin at multiple spatial scales; analyses were carried out within the R environment [49]. The spatial grid, where tracking locations and environmental data were overlaid, was based on the geographic limits of the tracking data (from 10°W to 110°W and from 40°S to 75°S). The 0.04° cell size (4 km^2^–840×2400 cells) was chosen according to the available oceanographic data (Table 1) and the accuracy of the tracking devices. We used a hierarchical modelling approach to identify those environmental parameters (see details in Table 1) that most accurately reflected the seascape and foraging habitats of king penguins. Prior to modelling, all environmental variables were standardized [50]. Strongly ‘correlated’ (|rs| >0.5) variables were identified by estimating all pair-wise Spearman rank correlation coefficients.

**Table 1:** Candidate environmental variables used for habitat modelling.

Explanatory variables	Satellite	Range (min-max)	Description
Bathymetry (BAT, m)	ETOPO	0–7958	Coastal versus pelagic domains
BAT gradient (BAT.G3, %)	ETOPO	0–100	Presence of topographic features
Chlorophyll a (CHLa, mg m^−3^)	Aqua/MODIS	0–24.91	Productivity
CHLa gradient (CHLa.G3, %)	Aqua/MODIS	0–100	Frontal systems
Sea Surface Temperature (SST, °C)	Aqua/MODIS	0–21.32	Temperature of the ocean’s surface
SST gradient (SST.G3, %)	Aqua/MODIS	0–100	Frontal systems
Sea Surface Height (SSH, cm)	AVISO	46.30–51.29	Mean sea level variation
SSH gradient (SSH.G3, %)	AVISO	0–100	Frontal systems

Dynamic variables were available on a monthly basis. As the variables differed in their spatial resolutions, they were aggregated to match a standard grid with cell size of 0.04°. Spatial gradients were estimated as their proportional change (PC) within a surrounding 3×3 cell (0.75°×0.75°) grid using a moving window as follows: PC = ((maximum value – minimum value)×100)/(maximum value).

Only positional data obtained between 1 January 2008 and 31 March 2008 were used for GLM modelling, because before this period the different locations of the breeding sites and subsequently the immediate surrounding oceanography may have influenced the analysis. The onset of this period was chosen to allow birds to move, following their departure, sufficiently far from the colony to avoid this influence (based on a mean daily distance of 45 km per day; see Table 2). Also, due to the cessation of some satellite transmitters, the number of data obtained after this period were not sufficient to be applied in a GLM model. In total we obtained 640 cells with presence of king penguins from South Georgia and 579 with presence of king penguins from the Falkland Islands. To obtain binomial response variables we followed the method developed by [51] and generated the same number of pseudo-absences as presences. To this end, we followed several rules to ensure that the pseudo-absences were located inside the surveyed areas but not in areas that are known to be suitable areas for penguins (we masked the surrounding of each presence data, using a moving window of 3×3 grid cells). We also generated 100 further sets of pseudo-absence for model construction and validation. We fitted generalized lineal models (GLMs; [52]) using as response variable the presence/absence of king penguins in a 4-km^2^ cell using binomial errors and a logit link. To take into account the variability of the study area, we decided to use a resampling scheme to obtain a balanced sample [53], [54], randomly choosing the same number of cells with presence and with pseudo-absence. We reserved a random sample of 30% of cells with presence and 30% of cells with absence for model cross-validation and used the remaining 70% for model fitting. This procedure was repeated 100 times. In each repetition the cells with presence were the same (but a new cross-validation sample with replacement was obtained), while cells with absence were sampled without replacement. Variables for the models were selected from the initial set by a backward-forward stepwise procedure starting from a full model that included all potential variables. The Akaikés Information Criterion (AIC) was used to retain a term and select between candidate models [55]. We considered as competing models those for which the differences between AIC and the AIC of the best candidate model (the one with the smallest AIC) was Δi ≤2 [56]. For those models we also calculated the AIC weight wi, (the relative model likelihood), which assesses how much the model is supported by the data, relative to the set of competing models.

**Table 2:** Summary of parameters characterising the migratory behaviour of king penguins in the South Atlantic.

Penguin	Date ofdeparture	End oftransmission	Duration(days)	Maximumdistanceto colony(km)	Minimumdistancecovered(km)	Meandailydistance(km/day)	Maximumdailydistance(km/day)
*WaRu*	13.12.2007	29.01.2008	49	779	2438	51±28	134
*Gus*	14.12.2007	31.01.2008	51	668	1837	38±22	82
*Susi*	13.12.2007	03.02.2008	54	1032	2781	52±24	137
*RuWa*	13.12.2007	15.02.2008	66	960	3212	49±23	109
*Iona*	18.12.2007	22.02.2008	73	696	2848	43±27	94
*Caldera*	13.12.2007	07.03.2008	87	841	3096	36±22	98
*Hansueli*	18.12.2007	21.04.2008	132	1977	5286	42±22	106
*Leo*	14.12.2007	29.04.2008	140	4015	6320	46±24	106
*Jacki*	13.12.2007	12.05.2008	153	3132	6794	45±26	127
*Youngster*	13.12.2007	28.08.2008	261	4783	11712	45±23	107
**FI Mean ± SD**			**107±67**			**45±25**	
*Ueli*	15.12.2007	28.02.2008	77	847	2374	31±23	105
*Ursula*	16.12.2007	29.02.2008	78	661	3192	43±23	81
*Saanenland*	17.12.2007	13.03.2008	91	834	3602	41±23	113
*Tankini*	16.12.2007	04.04.2008	113	1130	5456	50±23	98
*Dixi*	15.12.2007	22.05.2008	161	2603	7386	46±28	124
*Traudel*	16.12.2007	03.06.2008	173	2648	9114	54±27	135
*Wicky*	20.12.2007	06.06.2008	176	2150	7570	45±22	105
*King Georg*	17.12.2007	06.06.2008	176	3445	6684	39±24	174
**SG Mean ± SD**			**131±45**			**45±24**	
**FI & SG Mean ± SD**			**117±58**			**45±25**	

Birds in the upper half originated from the Falkland Islands (FI), those in the lower half from South Georgia (SG). The minimum distance covered is the sum of all distances between consecutive positions and does not take into account deviations from a straight line course. The mean daily distance is given with standard deviation.

### Model Validation

Each time a data-set was generated, 70% of the data was used to build a model and the remaining 30% was reserved to validate it. The area-under-the-curve (AUC) of the receiver operating characteristic (ROC) plot was computed for each of the 100 replicate models with each set of validation data to estimate its predictive power through cross-validation [57]. The AUC ranges from 0 (when model discrimination is not better than random) to 1 (perfect discriminatory ability, [58]). Predictive models are considered usable if AUC≥0.7 [59].

### Mapping Predictions

We used the most parsimonious model to build a predictive map of current juvenile king penguin distribution in the Southern Ocean. To produce this map, we used the option in IDRISI Taiga [60] to export variables as a data matrix, applied the predict.gam procedure to make predictions on the new data matrix, and then exported the predicted values at the scale of the response back to IDRISI to produce a probability map.

## Results

### Dispersal of Juveniles

Apart from some individual variability described below, no general differences in the migratory behaviour of birds from either site were apparent. This applied also to the various migratory parameters calculated (Table 2). Overall, 18 king penguin juveniles were tracked for a total of 2,111 days, those from the Falkland Islands for 1066 days (n = 10) and those from South Georgia for 1045 days (n = 8). The mean tracking period was 117±58 days (range: 49–261 days) with no significant differences between birds from the Falkland Islands (107±67 days) and South Georgia (131±45 days). The maximum distance to the colony varied between 668 km and 4,783 km for penguins from the Falkland Islands and between 661 km and 3,445 km for birds from South Georgia. The minimum distance covered showed no significant differences. The mean daily distance covered was also similar, with 45±25 km in both cases (Falkland Islands 45±24 km; South Georgia: 45±25 km). However, all birds, whether from the Falkland Islands or South Georgia, exhibited high levels of variability in the daily distances travelled. The majority of daily positions occurred within 10 km of the previous position; however, some were more than 100 km distant from the preceding position. Thus, birds sometimes travelled considerable distances from their previous position, and they subsequently occupied different water masses.

**Figure 1:**
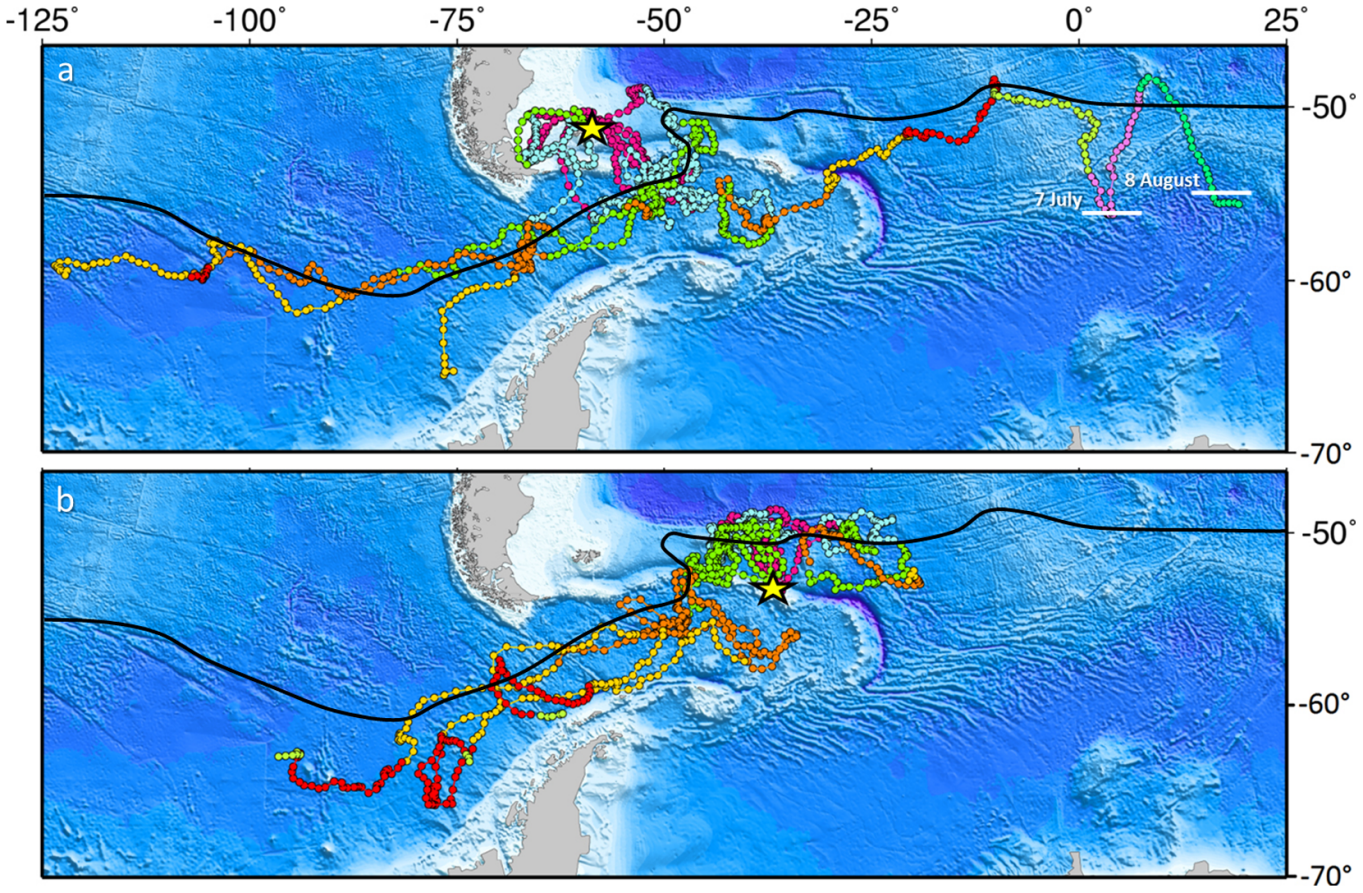
Satellite tracks obtained from juvenile king penguins from (a) the Falkland Islands and (b) South Georgia. Breeding sites are marked by a yellow asterisk. Track colours represent a monthly time scale, with positions in pink (December 2007), blue (January 2008), yellow-green (February), orange (March), golden (April), red (May), olive (June), violet (July) and green (August 2008). The black line indicates the approximate position of the Antarctic Polar Front. White lines in (a) indicate the edge of the winter pack ice at the date given.

During the first 20 days of deployment, seven birds from the Falkland Islands travelled directly south to the APF, while three birds (*Gus*, *Iona* and *Leo*) remained over the Falklands Plateau (Figure 1). In contrast, all birds from South Georgia showed a consistent pattern of behaviour and travelled directly north to the APF (Figure 1). After approximately 18 days after the deployment of tags, the birds from South Georgia switched to a different pattern of behaviour, which was consistent with that of the birds from the Falkland Islands.

Following this initial dispersal, the majority of birds from both study sites remained for varying time periods in the APF located half-way between the Falkland Islands and South Georgia, before seven of the eight birds tracked for periods for as much as 4 months migrated west into the Pacific Ocean until transmission ceased (Figure 1). Only one bird from the Falklands, *Youngster*, headed east and travelled into the Indian Ocean. During this movement, the bird turned south twice until reaching the edge of the winter pack ice before turning north and later east again (Figure 1). During the first trip south the bird arrived at the ice edge on 7 July and stayed for one week, the second time it arrived on 8 August but transmission ceased on 28 August.

### Environmental Parameters Influencing Juvenile Dispersal

**Figure 2:**
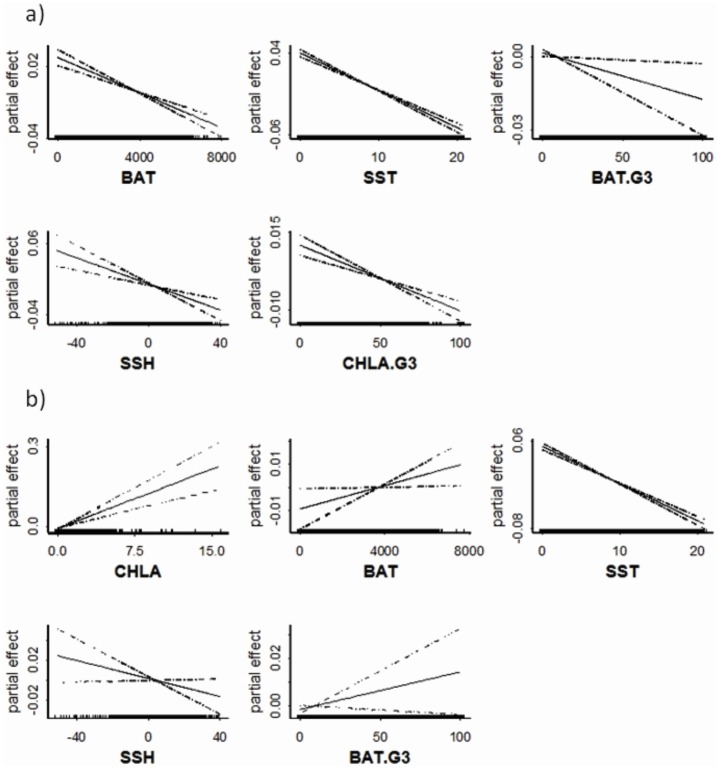
Mean partial effects (solid line) and standard error of the partial effect (broken lines) for the variables retained in the final generalized lineal model (GLM) of the probability of occurrence of king penguins from (a) the Falklands Islands and (b) South Georgia. Parameters are shown in the order of the stepwise selection by the GLM (Table 3).

**Figure 3:**
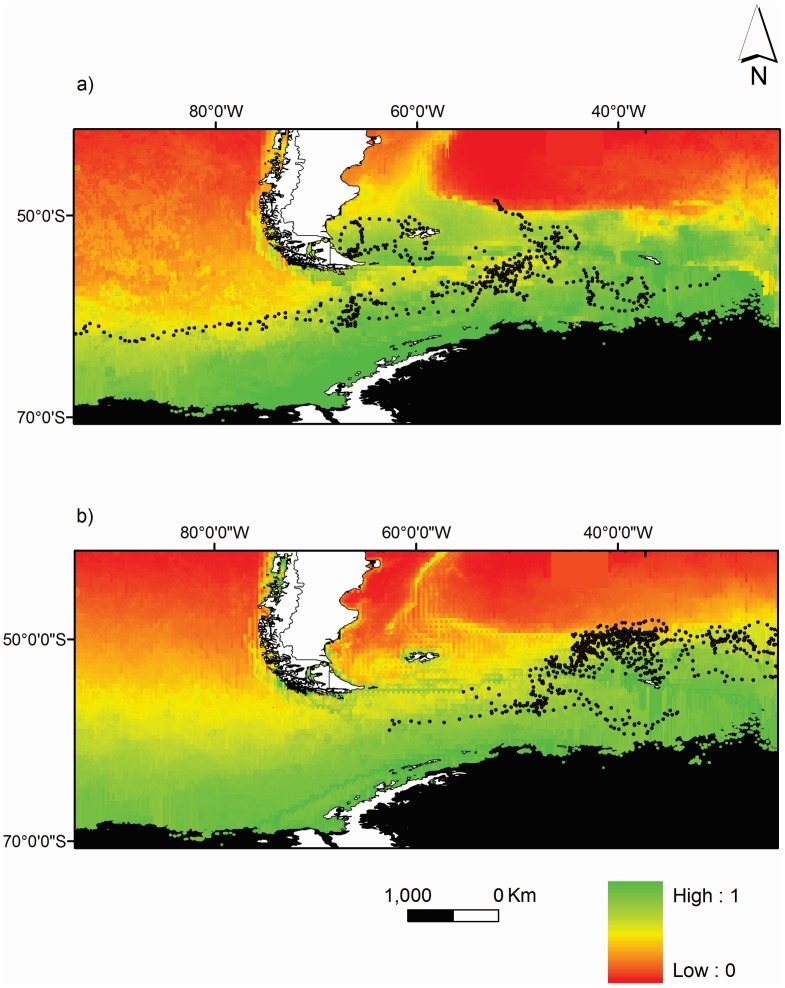
Distribution of king penguins from (a) the Falklands Island and (b) South Georgia in the southern oceans between 1 January and 31 March 2008. Values represent the probability of finding king penguins in a 4-km cell predicted by the best model (Table 3). Areas in black correspond to regions without predictions or outside the model’s environmental space. Daily positions of king penguins are indicated by circles.

The most parsimonious model for the probability of occurrence of king penguins from the Falkland Islands included the variables: Bathymetry, sea surface temperature, bathymetric gradient, sea surface height and chlorophyll a gradient (Table 3, Figure 2). This model indicated that king penguins from the Falkland Islands used relatively shallow areas of bathymetry and low bathymetric variability, low sea surface temperature and sea surface height and low chlorophyll a variability (Figure 3a). The most parsimonious model for the probability of occurrence of king penguins from South Georgia included the variables: Chlorophyll a, bathymetry, sea surface temperature, sea surface height and bathymetric gradient (Table 3). The best model showed chlorophyll a, bathymetry and bathymetric gradient had the strongest positive effect, and sea surface temperature and sea surface height a negative effect on the presence of king penguins from South Georgia. Therefore, this result suggests that king penguins from South Georgia selected relatively deeper waters with high concentrations of chlorophyll a, greater bathymetric variability and low sea surface temperature and sea surface height (Figure 3b).

**Table 3:** GLM models obtained by stepwise selection of habitat variables influencing king penguins from the Falkland Islands and South Georgia.

Code	GLM Models	AIC	Δ AIC	AUC ± SE
	***King penguins from the Falkland Islands***			
1	**−BAT −SST −BAT.G3 −SSH −CHLA.G3**	**548.06**	**0.00**	**0.80±0.02**
2	**−**BAT **−**SST **−**SSH **−**CHLA.G3	549.18	1.12	
	***King penguins from South Georgia***			
3	**+CHLA +BAT −SST +BAT.G3 −SSH**	**600.34**	**0.00**	**0.76±0.01**
4	+CHLA +BAT **−**SST **−**SSH	601.35	1.01	
5	+CHLA **−**SST –SSH	601.40	1.06	

Parameters are shown in the order of importance, derived from the stepwise selection. In addition, the plus or minus signs preceding the parameters indicate whether there is a positive or negative effect. For each competing model, the Akaike Information Criterion (AIC) and the difference between the AIC of the current model and the most-parsimonious model (ΔAIC) are given. The most parsimonious model for each case are marked in bold and mean area-under-the-curve (AUC) values computed for 100 replicate parameterizations of the models. All 100 replicates had AUC≥0.7.

### Model Validation and Predictive Cartography

**Figure 4:**
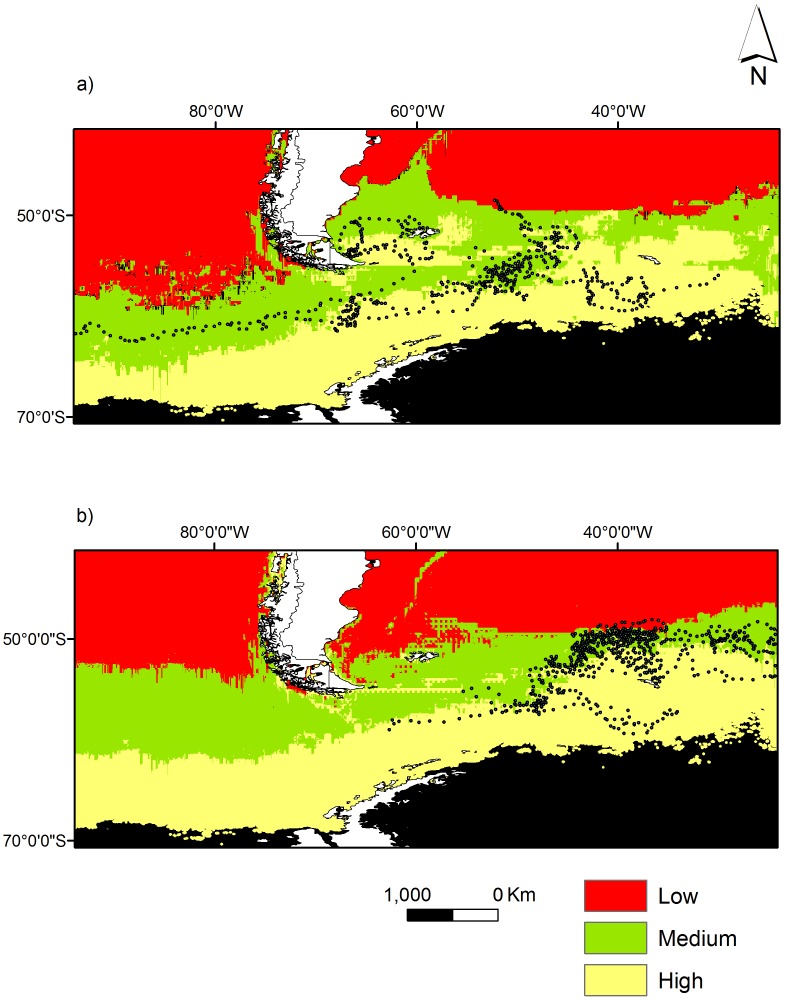
Predicted distribution of juvenile king penguins from (a) the Falklands Islands and (b) South Georgia in the southern oceans between 1 January and 31 March 2008. Values represent the probability of finding king penguins in a 4-km^2^ cell predicted by the best model (Table 3) and are categorized into three classes (low: <0.33, medium: 0.33–0.66, high: >0.66) to facilitate interpretation. Areas in black correspond to regions without predictions or those outside the model’s environmental space.

Predictive models fitted the data well, with a mean AUC of 0.80±0.02 for the king penguins from the Falkland Islands and 0.76±0.01 for the birds from South Georgia, which indicated that selected models were robust and considered potentially useful for predicting the distribution of juvenile king penguins within the ranges of predictor variables. The predictive map of the distribution of juvenile king penguins in the Southern Ocean, taking into account all the effects contained in the best general model is shown in Figure 4. While birds from both study sites have a high probability of occurring within waters south of the APF, there are slight differences in that juveniles from the Falkland Islands additionally have a high probability of occurring over the Patagonian Shelf between the Falkland Islands and the South American continent, while for South Georgia birds the probability of occurring between the natal colony and the APF to the north was higher than for Falkland Islands birds.

## Discussion

To our knowledge, this is the first study to investigate the post-natal dispersal of juvenile king penguins. Furthermore, spatial differences in the migratory behaviour were evaluated by simultaneously tracking birds from two breeding sites, which are subject to different climatic and oceanographic conditions.

### Potential Impact of Devices

A tracking period of up to 261 days and a horizontal displacement of up to c. 12,000 km represents the longest tracking duration for king penguins yet recorded in the literature and potentially even one of the longest when compared with studies on other non-flying, air-breathing marine vertebrates. While it is well accepted that the attachment of external devices, especially those having an antenna, can greatly influence the foraging performance and ultimately survival of marine predators [44], [46], we assume, for a number of reasons, that the behaviour of the birds studied was not seriously affected by these small and hydrodynamic devices. The cessation of transmissions after longer deployment periods is unlikely to have been caused by device technical failure and those that occurred during the initial period of tracking most probably happened because of device attachment failure, or because the birds perished, either because of starvation or predation. Afterwards, the cessation of transmissions most probably happened because of battery exhaustion. Minimum first-year survival for juvenile king penguins has been estimated to range between 40% and 50% [61], and it might reasonably be assumed that mortality rates of inexperienced birds are highest in the first weeks after leaving the colony. This situation could then account for our early failures; our results showed that 50% of instrumented birds were still tracked after 100 days. Further, the same methodology that we used has successfully been applied for periods greater than 3 months on a variety of smaller penguin species such as rockhopper (*Eudyptes chrysocome*) and magellanic (*Spheniscus magellanicus*) penguins [62], [63], [64], [65]. We therefore assume that the equipment of juvenile king penguins with small satellite transmitters did not severely impact upon the birds’ behaviour and survival.

### Habitat use and Colony Differences

The foraging range of adult king penguins during the breeding season is one of the most extensively studied among marine air-breathing vertebrates (review in [26]). Research has shown that adult king penguins are dependent on frontal zone features, irrespective of their breeding site and its relative position in relation to these features. In summer, they forage predominantly within the vicinity of the APF and, at some breeding sites, to a lesser extent, further north at the Sub-Antarctic front (Falkland Islands: [32], [66]; South Georgia: [30], [32], [67], [68]; Crozet Islands: [28], [29], [31], [32], [69]; Kerguelen Islands: [32], [70]; Heard Island: [71], but see also [33]; Macquarie Island [33], [72]) or further south at the Southern Antarctic Circumpolar Front (South Georgia: [73]).

Myctophid fish are abundant in the APF [74] and constitute the major prey of king penguins [35], [75]. In autumn and winter, however, adults provisioning chicks forage mainly further south in Antarctic waters between the APF and the northern pack-ice edge [34], [69], [71]; squid are also more important in the diet of king penguins during this time of the year [35], [36]. The only exception from this pattern is for adult king penguin breeding on the Falkland Islands, where in winter they make use of the slope of the Patagonian Shelf as far north as 38°S [32].

Our results indicate that juvenile birds only exhibited this foraging pattern during the initial phase of the tracking period, just after they left their natal colonies. For example, all birds from South Georgia, like adult breeding birds, moved northwards towards the APF. In contrast, three out of the ten birds studied from the Falkland Islands dispersed over the Patagonian Shelf between the Falkland Islands and the South American continent, presumably relying on different food items over the comparatively shallow shelf waters. The same area is also a favoured foraging habitat for rockhopper penguins from western colonies in the Falkland Islands [76] and from Staten Island, located off the southeastern tip of South America [63], as well as for magellanic penguins from Martillo Island in the Beagle Channel [65] during different times of the year. These differing migratory strategies may indicate that at least some juveniles from the Falkland Islands reacted to the different environmental conditions around the Falkland Islands and made use of the highly productive Patagonian Shelf area off the coast of Tierra del Fuego, which is also likely to be reflected in different prey items taken.

By early January at the latest, all birds had moved further south and dispersed within the vicinity of the APF, located half-way between the Falklands and South Georgia. Afterwards, juveniles remained close to the APF but moved further east and west. Thus, there was only a temporary overlap in the foraging areas used by adults and juveniles during the initial tracking period. As the season progressed into winter, their foraging areas may have overlapped again, but not necessarily only with adults from their site of origin. For example, in July and August *Youngster* used an area close to the pack-ice-edge which is also potentially frequented by birds from colonies located in the Indian Ocean [31], [34]. At the northern limit of the expanding winter pack ice, productivity is enhanced [77] and the myctophid fish *Electrona antarctica* and *Gymnoscopelus braueri* are abundant in the upper 500 m of the water column [78], thereby providing a suitable food resource to foraging king penguins and other marine predators such as, for example, juvenile emperor penguins [22]. Furthermore, and as mentioned earlier, squid may also play a major role as potential food for penguins at this time [35], [36], [79].

Thus, it appears that spatial segregation between juveniles and adults, which has been found in other seabird and marine mammal species in the Southern Ocean [7], [8], [22], [23], [80] limits the level of competition between younger birds and more experienced adults foraging in the vicinity of their breeding colony after the initial dispersal, thereby reducing intra-specific competition for food. However, once birds have begun to gain experience and the season progresses, the winter foraging areas in the vicinity of the APF and further south have not only to be shared with adults, but also with a number of other seabirds and marine mammals (e.g. [81]).

Remarkably, with the exception of *Youngster*, all birds tracked migrated in a westerly direction, against the direction of the prevailing current. These movements against the prevailing currents have also been observed in a pre-moult chinstrap penguin (*Pygoscelis antarctica*) [82] and a rockhopper penguin during winter [63], whereas during the breeding season rockhopper and magellanic penguins from the Falkland Islands were assumed to use the prevailing currents to reduce energy expenditure during foraging [83], [84]. The reasons for the movement against the prevailing currents remain purely speculative, but could be explained by olfactory cues to find areas of high productivity, as has been shown in several procelariiform seabirds and two species of penguins [85]. Furthermore, it remains unknown as to whether the birds maintained this direction and circumnavigated the Antarctic continent during their first year at sea, or whether they turned at some stage and headed back towards their region of origin, which has, for example, been observed in juvenile emperor penguins [22]. As birds do not necessarily return to their home colony to moult, juveniles can spend several years exploring the Southern Ocean. Usually, king penguins return to their natal colony upon reaching sexual maturity at the age of 5–6 years [25], [26], but in some instances they start breeding elsewhere. For example, a chick from South Georgia banded as a fledgling began to breed in the Falkland Islands about six years later ([86], Pütz, pers.obs.). Similarly, a chick from the same banding study at Husvik on South Georgia, was found breeding at the St Andrews Bay colony some 12 years later (Trathan, pers.obs.).

While the foraging areas in general were, apart from the initial phase, quite similar for juveniles from both study sites, some differences were apparent when applying the GLM model. Of course it cannot be ruled out that genetically predetermined adaptations in their foraging behaviour exist, but our results indicate that behavioural adaptations linked to their breeding site are developed over time, presumably supported by foraging in association with congeners and other species, which has been shown to be the case in juvenile brown boobies [87]. As this is likely to involve mostly non-breeding birds from different age classes, foraging in flocks presumably enables birds to ‘learn’ about prospective feeding places. In wandering albatrosses, the foraging behaviour of immatures was shown to be partly innate and partly learned over time, until the birds had acquired the foraging skills needed to breed successfully [10]. Our results indicate that a comparable development of foraging skills over time, including specific adaptations to environmental conditions, may also apply to king penguins. This is further substantiated by the fact that king penguins have been shown to adjust their diving behaviour with increasing age, thereby reducing their energy expenditure during foraging dives [88].
